# Preventive behaviors and behavioral risk factors among gynecologic cancer survivors: Results from the 2020 Behavioral Risk Factor Surveillance System Survey

**DOI:** 10.1002/cam4.6134

**Published:** 2023-06-30

**Authors:** Steven S. Coughlin, Biplab Datta, Justin Xavier Moore, Marlo M. Vernon, Martha S. Tingen

**Affiliations:** ^1^ Department of Population Health Sciences Augusta University Augusta Georgia USA; ^2^ Institute of Public and Preventive Health, Augusta University Augusta Georgia USA; ^3^ Cancer Prevention, Control, & Population Health Program, Georgia Cancer Center Augusta University Augusta Georgia USA; ^4^ Department of Medicine, Medical College of Georgia Augusta University Augusta Georgia USA

**Keywords:** cervical cancer, ovarian cancer, physical activity, preventive behaviors, rural residence, smoking, uterine cancer

## Abstract

**Background:**

Maintaining a healthy lifestyle is an important factor in promoting positive outcomes for gynecologic cancer survivors.

**Methods:**

We examined preventive behaviors among gynecologic cancer survivors (*n* = 1824) and persons without a history of cancer in a cross‐sectional analysis, using data from the 2020 Behavioral Risk Factor Surveillance System survey (BRFSS). BRFSS is a cross‐sectional telephone‐based survey of U.S. residents 18 years of age and older, which collects information about health‐related factors and use of preventive services.

**Results:**

The prevalence rates of colorectal cancer screening were respectively 7.9 (95% CI: 4.0–11.9) and 15.0 (95% CI: 4.0–11.9) %‐points higher among gynecologic and other cancer survivors compared to that of 65.2% among those without any history of cancer. However, no differences were observed in breast cancer screening between gynecologic cancer survivors (78.5%) and respondents without any history of cancer (78.7%). Coverage of influenza vaccination among gynecologic cancer survivors were 4.0 (95% CI: 0.3–7.6) %‐points higher than that of the no cancer group, but 11.6 (95% CI: 7.6–15.6) %‐points lower than that of the other cancer group. Pneumonia vaccination rate among gynecologic cancer survivors, however, was not statistically different than that of other cancer survivors and respondents with no history of cancer. When examining modifiable risk behaviors, the prevalence of smoking among gynecologic cancer survivors was 12.8 (95% CI: 9.5–16.0) and 14.2 (95% CI: 10.8–17.7) %‐points higher than smoking prevalence among other cancer survivors and respondents without any history of cancer. The rate differentials were even higher in rural areas, 17.4 (95% CI: 7.2–27.6) and 18.4 (95% CI: 7.4–29.4) %‐points respectively. There were no differences in the prevalence of heavy drinking across the groups. Lastly, gynecologic and other cancer survivors were less likely to be physically active (Δ = −12.3, 95% CI: −15.8 to −8.8 and Δ = −6.9, 95% CI: −8.5 to −5.3, respectively) than those without any history of cancer.

**Conclusion:**

Smoking prevalence among gynecologic cancer survivors is alarmingly high. Intervention studies are needed to identify effective ways to assist gynecologic cancer survivors to quit smoking and refrain from hazardous alcohol consumption. In addition, women with gynecologic malignancies should made aware of the importance of physical activity.

## Abstract 2

Maintaining a healthy lifestyle is an important factor in promoting positive outcomes for gynecologic cancer survivors. We examined preventive behaviors among gynecologic cancer survivors in a cross‐sectional analysis, using data from the 2020 Behavioral Risk Factor Surveillance System. Smoking prevalence among gynecologic cancer survivors is alarmingly high. Intervention studies are needed to identify effective ways to assist gynecologic cancer survivors to quit smoking and refrain from alcohol consumption.

## 1 INTRODUCTION

As a result of improvement in therapies and supportive care, the prevalence of gynecologic (uterine, cervical, and ovarian) cancer survivors continue to grow over the past few decades. Uterine cancer is the fourth most common cancer among women in the U.S., with an estimated 66,570 new cases in 2021.^1^ There were an estimated 14,480 new cases of cervical cancer and an estimated 21,410 new cases of ovarian cancer in the U.S. in 2021.^1^ As of January 1, 2019, it is estimated that there were 807,860 uterine cancer survivors, 283,120 cervical cancer survivors, and 249,230 ovarian cancer survivors in the U.S.^2^

Maintaining a healthy lifestyle is an important factor in promoting positive outcomes for gynecologic cancer survivors.^3^, ^4^, ^5^ Health behaviors, such as engaging in physical activity, not consuming large amounts of alcohol, abstaining from cigarette smoking, receiving routine immunizations, and undergoing recommended cancer screening tests, is known to improve health and quality of life.^6^, ^7^ For example, physical activity, healthy nutrition options, maintaining a healthy weight, and avoiding smoking is associated with reduced risk of cancer recurrence, secondary cancers, and chronic illness (e.g., obesity, cardiovascular disease, diabetes).^8^, ^9^

Gynecologic cancer survivors who have completed primary therapy for the disease often have complex health care needs that include close follow‐up to detect recurrence and second primary malignancies, and the management of cancer‐related morbidities.^10^ Appropriate care during the post‐initial treatment survivorship phase includes preventive care visits and screening for cancer recurrence.^11^, ^12^, ^13^

Survivors of gynecologic cancer who live in rural areas often experience several barriers to receiving appropriate follow‐up care. Residents of rural areas are more likely to be low‐income and to lack health insurance than residents of urban areas.^14^ Rural residence has been related to health disparities and greater mortality risk in gynecologic cancer patients.^15^ However, there is a need for more studies of gynecologic cancer survivors in rural areas.

We examined preventive behaviors and modifiable behavioral risk factors among gynecologic cancer survivors, using data from the cancer survivorship module of the 2020 Behavioral Risk Factor Surveillance System (BRFSS). This manuscript considers survivors of gynecologic cancer from the time of diagnosis and initiation of treatment. We compared behavioral risk factors and preventive measures among gynecologic cancer survivors, female survivors of other types of cancer, and women without a history of cancer. Based on the existing evidence supporting greater likelihood of screening service utilization among cancer survivors,^16^, ^17^ our hypothesis was that the prevalence of preventive measures among gynecologic cancer survivors would be that of survivors of other types of cancer and higher than the prevalence among women without a history of cancer. In addition, in line with the existing evidence on rural–urban differences in health behaviors and outcomes,^18^, ^19^ we hypothesized that the prevalence of preventive measures and behavioral risk factors would vary by urban–rural residence.

## 2 METHODS

BRFSS is a cross‐sectional telephone‐based survey of U.S. residents 18 years of age and older, which collects information about health‐related factors and use of preventive services. Our sample consisted of 110,820 adult (aged ≥18 years) women from 28 states (Arizona, Connecticut, Delaware, Georgia, Hawaii, Indiana, Kansas, Louisiana, Massachusetts, Maryland, Maine, Michigan, Missouri, Mississippi, Montana, North Carolina, Nebraska, New Jersey, New Mexico, New York, Ohio, Oklahoma, Rhode Island, South Dakota, Utah, Virginia, Vermont, Wisconsin) who completed information on types of cancer under the cancer survivorship module in the 2020 BRFSS. The mean and median weighted response rate (landline and cell phone combined) for this group of states were 47.7% and 46.8%, respectively.

We assessed four preventive health measures–colorectal cancer screening (as per the U.S. Preventive Services Task Force recommendation) among women aged 45–75 years,^20^ breast cancer screening (mammogram in past 2 years) among women aged 50–74 years,^20^ vaccination for influenza, and vaccination for pneumonia among women age ≥65 years; and three behavioral risk factors–smoking, heavy drinking, and no leisure time physical activity. A respondent was determined to be a current smoker if she smoked at least 100 cigarettes in her entire life and smoked every day or some days at the time of the survey. Heavy drinking was defined as having more than 14 drinks per week for men and more than 7 drinks per week for women. Physical activity was defined as participation in physical activities or exercises other than regular job during the past 30 days preceding the survey. Of note, we did not have information on physical activities in the workplace; neither do we know about the intensity of the activity.

We divided the sample into three mutually exclusive groups–no history of cancer (other than non‐melanoma skin cancer), gynecologic cancer, and other cancer (other than non‐melanoma skin cancer). Of note, respondents who reported to have more than one type of cancer (including non‐melanoma skin cancer) were grouped based on their most recent diagnosis of cancer. As such, there could be overlaps for some observations.

We examined how the prevalence of preventive health behaviors and behavioral risk factors vary across these groups. We fitted separate univariate and multivariable logistic regression models for each of the seven preventive measures and behavioral risk factors to obtain the unadjusted and adjusted odds in favor of complying with the preventive measure or having the risk factor for being a gynecologic cancer survivor or other cancer survivor compared to someone with no history of cancer. Our outcome variables are binary variables indicative of respective preventive measures or risk factors. Our key explanatory variables are dichotomous variables indicating gynecologic or other cancer survivorship; no history of cancer serves as the reference category. In the multivariable model we controlled for age (18–49 years [base category], 50–64 years, and ≥65 years); race and ethnicity (White [base category], Black, Hispanic, and other); educational attainment (less than high diploma [base category], high school graduate, some college, and college graduate); household income (less than $15,000 [base category], $15,000 to $24,999, $25,000 to $34,999, $35,000 to $49,999, and $50,000 or more); marital status (not married [base category], married, and divorced/ widowed/separated); employment status (employed [base category]–“employed for wages or self‐employed”, not employed–“out of work for <1 year or ≥1 year, homemaker, student, and unable to work”, and retired); and fixed effects. Of note, in the multivariable model, we did not evaluate the sociodemographic predictors of preventive behaviors or risk factors. Rather we intended to examine how gynecologic cancer status was associated with certain behaviors after accounting for the socio‐demographic correlates. Observations, for which sociodemographic data were missing, were excluded from the multivariable specification.

To check the robustness of our analysis, we further estimated the models for urban and rural sub‐groups. Urban consists of counties that are classified as large central metro, large fringe metro, medium metro, small metro, and micropolitan statistical areas.^21^ Rural consists of noncore counties outside of the micropolitan statistical areas. Additionally, to explore the relationships across different types of gynecologic cancer, we estimated the models (for full sample only) with the key explanatory variable having the following mutually exclusive categories: no history of cancer (base category), other cancer, cervical cancer, endometrial cancer, and ovarian cancer. The statistical analyses were conducted using Stata 17.0 and all estimates account for complex survey weights. Level of significance was set at 5% level.

This study utilized BRFSS public use data that include no personal identifiers and was therefore except from review by the Augusta University Institutional Review Board.

## 3 RESULTS

Of the 110,820 women in our sample, 1824 were gynecologic cancer survivors, and 9843 were survivors of other types of cancer. Around 90% of the women in the sample had no history of cancer. Among respondents having gynecologic cancer, 904 had cervical‐, 556‐ had endometrial‐, and 364 had ovarian‐ cancer. The weighted prevalence of gynecologic cancer was higher in rural areas than in urban areas (Δ = 0.75%‐points, 95% CI: 0.32–1.19). Table 1 shows the demographic and socioeconomic characteristics of the study participants by mutually exclusive categories and urban and rural residence. While the largest weighted share (37.35%) of gynecologic cancer survivors in the urban areas were elderly (age ≥ 65 years), the largest weighted share (38.37%) in rural areas were younger adults (age 18–49).

**TABLE 1:** Demographic and socioeconomic characteristics of study participants by urban–rural residence and type of cancer.

	No history of cancer *N* [%]		Other cancer *N* [%]		Gynecologic cancer *N* [%]		Gynecologic cancer type *N* [%]		
	Urban	Rural	Urban	Rural	Urban	Rural	Cervical	Endometrial	Ovarian
*N*	85,713	13,440	8315	1528	1517	307	904	556	364
Age									
18–49	33,109	3987	714	93	352	68	303	46	71
	[51.85]	[42.52]	13	[9.64]	[31.15]	[38.37]	[44.45]	[13.22]	[26.25]
50–64	23,913	4032	2195	366	452	100	297	148	107
	[25.85]	[29.55]	[31.65]	[26.82]	[31.5]	[30.51]	[30.6]	[28.95]	[36.82]
65+	28,691	5421	5406	1069	713	139	304	362	186
	[22.3]	[27.93]	[55.35]	[63.54]	[37.35]	[31.12]	[24.95]	[57.83]	[36.93]
Race									
White	60,133	10,785	6979	1341	1219	251	700	479	291
	[63.34]	[80.45]	[81.72]	[87.27]	[79.92]	[84.92]	[77.76]	[87.23]	[77.78]
Black	8975	781	501	63	79	7	49	17	20
	[15.27]	[8.83]	[8.48]	[8.42]	[5.98]	[1.42]	[6.00]	[3.90]	[6.47]
Hispanic	7668	391	274	19	90	6	65	14	17
	[11.34]	[3.92]	[4.1]	[1.17]	[6.34]	[3.02]	[8.36]	[1.87]	[5.76]
Other	8937	1483	561	105	129	43	90	46	36
	[10.05]	[6.8]	[5.7]	[3.14]	[7.76]	[10.64]	[7.88]	[7.01]	[1.00]
Education									
<High school	5062	943	410	113	122	30	92	31	29
	[9.43]	[13.17]	[8.56]	[14.34]	[12.44]	[13.96]	[13.87]	[9.09]	[14.29]
High school	21,338	4151	2131	446	395	88	267	121	95
	[25.51]	[32.56]	[27.25]	[32.09]	[26.07]	[31.52]	[29.65]	[23.31]	[23.71]
Some college	24,403	4015	2392	504	489	107	305	184	107
	[31.31]	[32.63]	[30.49]	[34.62]	[36.28]	[41.54]	[36.3]	[40.15]	[33.55]
College	34,502	4288	3363	464	509	82	238	220	133
	[33.75]	[21.64]	[33.69]	[18.96]	[25.21]	[12.97]	[20.19]	[27.44]	[28.45]
Income									
<$15,000	5798	1166	546	142	156	42	115	46	37
	[9.04]	[12.55]	[9.58]	[12.3]	[14.37]	[14.4]	[15.46]	[12.46]	[14.07]
$15,000 to $24,999	10,637	2099	1145	251	267	62	195	78	56
	[15.87]	[21.97]	[17.57]	[26.15]	[20.07]	[24.15]	[22.99]	[17.44]	[18.18]
$25,000 to $34,999	6727	1264	748	165	144	40	90	60	34
	[9.56]	[11.71]	[10.15]	[11.4]	[10.9]	[15.26]	[12.12]	[12.66]	[7.82]
$35,000 to $49,999	9154	1567	927	174	168	39	106	65	36
	[13.06]	[15.01]	[13.27]	[13.26]	[13.18]	[16.17]	[13.82]	[12.66]	[13.8]
$50,000+	34,391	4246	3095	417	512	75	260	196	131
	[52.48]	[38.76]	[49.43]	[36.89]	[41.48]	[30.01]	[35.61]	[44.78]	[46.12]
Marital status									
Not married	18,157	2026	788	86	237	25	136	70	56
	[28.25]	[21.05]	[10.66]	[6.09]	[16.74]	[12.08]	[19.12]	[10.42]	[17.11]
Married	41,373	7083	3774	750	621	155	381	252	143
	[48.43]	[52.55]	[49.74]	[53.26]	[46.77]	[62.93]	[46.64]	[55.52]	[43.30]
Divorced/widowed/separated	25,261	4233	3706	687	647	126	380	230	163
	[23.32]	[26.4]	[39.6]	[40.65]	[36.49]	[24.99]	[34.24]	[34.05]	[39.59]
Employment									
Employed	40,357	5997	2231	345	503	103	334	150	122
	[52.34]	[47.64]	[30.94]	[22.48]	[33.78]	[40.34]	[38.07]	[26.51]	[36.49]
Not employed	19,828	2913	1527	302	435	92	320	105	102
	[28.28]	[28.7]	[22.64]	[23.54]	[34.01]	[31.45]	[41.17]	[21.36]	[32.42]
Retired	23,850	4328	4518	875	574	109	245	299	139
	[19.38]	[23.66]	[46.42]	[53.98]	[32.2]	[28.21]	[20.76]	[52.13]	[31.10]

*Note*: Percentages were obtained using complex survey weights. Data on education, income, marital status, and employment status were missing for 473, 24,656, 1085, and 1933 observations, respectively.

The weighted prevalence of colorectal cancer screening was significantly higher among gynecologic and other cancer survivors compared to those without any history of cancer. However, the weighted prevalence was lower among gynecologic cancer survivors compared to other cancer survivors, particularly in urban areas. A similar pattern was seen for influenza vaccination (Table 2). For breast cancer screening, no differences were observed between gynecologic cancer survivors and respondents without any history of cancer. Pneumonia vaccination was higher among both gynecologic and other cancer survivors compared to those without a cancer history. However, no statistically significant differences were observed across these two groups. On the other hand, the weighted prevalence of smoking was significantly higher among gynecologic cancer survivors, particularly in rural areas. There were no significant differences in the weighted prevalence of heavy drinking across the groups. A total of 25.97% of gynecologic cancer survivors were current cigarette smokers, whereas the smoking prevalence among other cancer survivors and respondents with no history of cancer was 11.76% and 13.22%, respectively. Among gynecologic cancer survivors, 5.08% were heavy alcohol drinkers, which was 5.54% and 6.30%, respectively, among survivors of other cancer and respondents without any history of cancer. Lastly, both gynecologic and other cancer survivors were less likely to be physically active than those without any history of cancer, particularly in urban areas.

**TABLE 2:** Prevalence of preventive health measures and behavioral risk factors among gynecologic cancer survivors, survivors of other types of cancer, and respondents without cancer by urban–rural residence.

	Percentage of women (%)					
	No history of cancer	Other cancer	Gynecologic cancer	Cervical cancer	Endometrial cancer	Ovarian cancer
Cancer screening						
Colorectal (age 45–75)						
All	*N* = 51,036	*N* = 6310	*N* = 1202	*N* = 563	*N* = 410	*N* = 229
	65.16	80.15	73.11	66.91	79.72	75.54
	(64.49, 65.83)	(78.54, 81.76)	(69.22, 77.00)	(60.76, 73.07)	(74.02, 85.42)	(67.02, 84.07)
Urban	*N* = 43,430	*N* = 5319	*N* = 1007			
	65.28	80.39	73.45			
	(64.57, 65.99)	(78.69, 82.10)	(69.32, 77.58)			
Rural	*N* = 7606	*N* = 991	*N* = 195			
	63.70	77.51	69.78			
	(61.76, 65.63)	(73.19, 81.82)	(58.89, 80.67)			
Mammogram (age 50–74)						
All	*N* = 43,444	*N* = 5634	*N* = 1048	*N* = 479	*N* = 370	*N* = 199
	78.72	81.70	78.52	74.16	81.70	82.15
	(78.10, 79.34)	(80.07, 83.34)	(74.86, 82.17)	(68.38, 79.93)	(75.77, 87.63)	(74.57, 89.73)
Urban	*N* = 36,792	*N* = 4748	*N* = 876			
	79.17	81.89	78.60			
	(78.52, 79.82)	(80.17, 83.61)	(74.70, 82.50)			
Rural	*N* = 6652	*N* = 886	*N* = 172			
	73.46	79.63	77.70			
	(71.61, 75.31)	(75.07, 84.20)	(68.80, 86.60)			
Vaccination						
Influenza						
All	*N* = 92,392	*N* = 9807	*N* = 1819	*N* = 901	*N* = 555	*N* = 363
	50.62	66.18	54.58	48.75	67.15	51.72
	(50.09, 51.14)	(64.58, 67.78)	(50.96, 58.20)	(43.68, 53.82)	(60.89, 73.40)	(43.47, 59.96)
Urban	*N* = 79,664	*N* = 8282	*N* = 1513			
	50.74	66.51	54.93			
	(50.19, 51.29)	(64.81, 68.20)	(51.08, 58.79)			
Rural	*N* = 12,728	*N* = 1525	*N* = 306			
	48.96	62.58	51.58			
	(47.30, 50.62)	(58.20, 66.97)	(41.44, 61.72)			
Pneumonia (age 65+)						
All	*N* = 31,130	*N* = 6317	*N* = 831	*N* = 294	*N* = 355	*N* = 182
	72.95	78.63	76.8	72.53	79.54	77.97
	(72.12, 73.79)	(76.88, 80.39)	(72.22, 81.38)	(64.07, 81.00)	(73.07, 86.02)	(68.63, 87.31)
Urban	*N* = 26,115	*N* = 5274	*N* = 694			
	73.27	78.83	75.85			
	(72.39, 74.15)	(76.95, 80.70)	(70.93, 80.76)			
Rural	*N* = 5015	*N* = 1043	*N* = 137			
	73.27	78.83	75.85			
	(72.39, 74.15)	(76.95, 80.70)	(70.93, 80.76)			
Tobacco/ alcohol use						
Current smoking						
All	*N* = 93,524	*N* = 9777	*N* = 1810	*N* = 898	*N* = 551	*N* = 361
	13.22	11.76	25.97	37.97	10.94	17.04
	(12.86, 13.57)	(10.64, 12.87)	(22.70, 29.24)	(32.98, 42.97)	(6.56, 15.32)	(10.80, 23.27)
Urban	*N* = 80,705	*N* = 8263	*N* = 1507			
	12.86	11.27	24.85			
	(12.49, 13.23)	(10.12, 12.41)	(21.40, 28.29)			
Rural	*N* = 12,819	*N* = 1514	*N* = 303			
	18.08	17.13	35.49			
	(16.77, 19.39)	(12.80, 21.47)	(25.39, 45.59)			
Heavy drinking						
All	*N* = 91,391	*N* = 9690	*N* = 1802	*N* = 893	*N* = 550	*N* = 359
	6.3	5.54	5.08	6.22	2.38	5.93
	(6.05, 6.55)	(4.73, 6.35)	(3.50, 6.66)	(3.91, 8.52)	(0.83, 3.94)	(1.35, 10.51)
Urban	*N* = 78,844	*N* = 8186	*N* = 1499			
	6.36	5.69	5.37			
	(6.10, 6.63)	(4.82, 6.57)	(3.63, 7.10)			
Rural	*N* = 12,547	*N* = 1504	*N* = 303			
	5.42	3.87	2.6			
	(4.67, 6.16)	(2.12, 5.62)	(0.07, 5.27)			
Physical activity						
Physically active						
All	*N* = 98,995	*N* = 9827	*N* = 1821	*N* = 904	*N* = 554	*N* = 363
	75.69	68.79	63.36	64.13	63.20	61.63
	(75.25, 76.13)	(67.24, 70.34)	(59.89, 66.83)	(59.39, 68.88)	(56.85, 69.55)	(53.35, 69.91)
Urban	*N* = 85,581	*N* = 8301	*N* = 1514			
	76.03	68.93	63.32			
	(75.57, 76.49)	(67.29, 70.57)	(59.61, 67.03)			
Rural	*N* = 13,414	*N* = 1526	*N* = 307			
	71.01	67.25	63.7			
	(69.55, 72.47)	(62.94, 71.55)	(54.17, 73.23)			

*Note*: Estimates were obtained using complex survey weights. 95% confidence intervals are in parenthesis. The three columns in the right are sub‐categories of gynecologic cancer.

Among gynecologic cancer survivors, colorectal cancer screening was significantly higher among endometrial cancer survivors compared to survivors of cervical cancer. While there was no significant difference in flu vaccination rates between survivors of cervical‐ and ovarian‐ cancer, the rate was significantly higher among survivors of endometrial cancer. Tobacco‐use, on the other hand, was significantly higher among cervical cancer survivors compared to other endometrial‐ and ovarian‐ cancer survivors. Though no differences in rates of heavy drinking were found between respondents without any history of cancer and gynecologic cancer survivors as a whole, the rate was significantly lower among survivors of endometrial cancer. No statistically significant differences were observed for mammogram, pneumonia vaccination, and leisure time physical activity across the three types of gynecologic cancer (Table 2).

The unadjusted and adjusted odds ratios in favor of preventive health measures and behavioral risk factors are presented in Table 3. The odds of being screened for colorectal cancer for gynecologic cancer survivors were 1.5 (95% CI: 1.2–1.8) times that of those without any history of cancer. The odds of receiving influenza (OR = 1.2, 95% CI: 1.0–1.4) and pneumonia (OR = 1.9, 95% CI: 1.7–2.3) vaccination were also higher among gynecologic cancer survivors compared to women without cancer. The odds of receiving a mammogram for gynecologic cancer survivors, however, were not different (OR = 1.0, 95% CI: 0.8–1.2) than those with no history of cancer. Compared to individuals without any history of cancer, the odds of smoking were significantly higher among gynecologic cancer survivors (OR = 2.3, 95% CI: 1.9–2.7) and significantly lower among survivors of other types of cancer (OR = 0.88, 95% CI: 0.78–0.98). In rural areas, the odds of current smoking for gynecologic cancer survivors were 2.5 times (95% CI: 1.59–3.92) that of those with no history of cancer. The odds of heavy drinking, on the other hand, were not statistically different from zero (OR = 0.8, 95% CI: 0.6–1.1). Lastly, the odds of physical exercise were significantly lower among both gynecologic (OR = 0.56, 95% CI: 0.48–0.65) and other cancer survivors (OR = 0.71, 95% CI: 0.66–0.76) compared to their peers without any history of cancer, particularly in urban areas. Compared to respondents with no history of cancer, survivors of other types of cancer had higher odds of colorectal cancer screening, having a mammogram, influenza and pneumonia vaccination, and lower odds of smoking and physical activity. The adjusted odds ratios were also very similar with varying magnitudes across different preventive measures and behaviors.

**TABLE 3:** Unadjusted and adjusted odds ratios in favor of preventive measures and behavioral risk factors for gynecologic cancer survivors and other cancer survivors.

	Unadjusted odds ratio			Adjusted odds ratio		
	No cancer	Other cancer	Gynecologic cancer	No cancer	Other cancer	Gynecologic cancer
Cancer screening						
Colorectal cancer						
All	1.000	2.160***	1.454***	1.000	1.759***	1.354*
	(Ref.)	(1.944, 2.400)	(1.190, 1.776)	(Ref.)	(1.547, 2.001)	(1.066, 1.720)
Urban	1.000	2.181***	1.471***	1.000	1.785***	1.364*
	(Ref.)	(1.949, 2.440)	(1.188, 1.822)	(Ref.)	(1.559, 2.044)	(1.052, 1.768)
Rural	1.000	1.964***	1.316	1.000	1.516*	1.212
	(Ref.)	(1.512, 2.551)	(0.780, 2.220)	(Ref.)	(1.099, 2.091)	(0.671, 2.187)
Mammogram						
All	1.000	1.207***	0.988	1.000	1.241***	0.991
	(Ref.)	(1.076, 1.355)	(0.793, 1.231)	(Ref.)	(1.090, 1.414)	(0.779, 1.263)
Urban	1.000	1.190**	0.966	1.000	1.231***	0.969
	(Ref.)	(1.052, 1.344)	(0.763, 1.222)	(Ref.)	(1.072, 1.413)	(0.746, 1.258)
Rural	1.000	1.413*	1.259	1.000	1.357	1.270
	(Ref.)	(1.050, 1.901)	(0.747, 2.121)	(Ref.)	(0.960, 1.918)	(0.697, 2.312)
Vaccination						
Influenza						
All	1.000	1.909***	1.172**	1.000	1.259***	1.038
	(Ref.)	(1.772, 2.057)	(1.012, 1.359)	(Ref.)	(1.153, 1.376)	(0.873, 1.234)
Urban	1.000	1.928***	1.183*	1.000	1.258***	1.013
	(Ref.)	(1.781, 2.087)	(1.011, 1.385)	(Ref.)	(1.146, 1.382)	(0.842, 1.217)
Rural	1.000	1.744***	1.110	1.000	1.279*	1.294
	(Ref.)	(1.429, 2.127)	(0.740, 1.666)	(Ref.)	(1.001, 1.634)	(0.811, 2.063)
Pneumonia						
All	1.000	3.076***	1.947***	1.000	1.565***	1.407***
	(Ref.)	(2.857, 3.312)	(1.681, 2.254)	(Ref.)	(1.411, 1.736)	(1.164, 1.702)
Urban	1.000	3.058***	1.920***	1.000	1.548***	1.343**
	(Ref.)	(2.828, 3.308)	(1.643, 2.244)	(Ref.)	(1.386, 1.728)	(1.096, 1.646)
Rural	1.000	3.246***	2.148***	1.000	1.732***	2.075**
	(Ref.)	(2.651, 3.974)	(1.457, 3.166)	(Ref.)	(1.318, 2.275)	(1.280, 3.365)
Tobacco/alcohol consumption						
Current smoking						
All	1.000	0.875**	2.303***	1.000	0.964	1.989***
	(Ref.)	(0.782, 0.978)	(1.938, 2.738)	(Ref.)	(0.844, 1.100)	(1.625, 2.435)
Urban	1.000	0.860*	2.240***	1.000	0.921	1.869***
	(Ref.)	(0.763, 0.969)	(1.857, 2.702)	(Ref.)	(0.801, 1.060)	(1.498, 2.331)
Rural	1.000	0.937	2.493***	1.000	1.495**	2.705***
	(Ref.)	(0.683, 1.286)	(1.586, 3.917)	(Ref.)	(1.018, 2.196)	(1.658, 4.413)
Heavy drinking						
All	1.000	0.872	0.796	1.000	0.939	0.930
	(Ref.)	(0.742, 1.025)	(0.572, 1.107)	(Ref.)	(0.780, 1.130)	(0.659, 1.313)
Urban	1.000	0.888	0.835	1.000	0.942	0.976
	(Ref.)	(0.750, 1.051)	(0.591, 1.178)	(Ref.)	(0.776, 1.143)	(0.682, 1.399)
Rural	1.000	0.703	0.466	1.000	0.922	0.522
	(Ref.)	(0.429, 1.150)	(0.161, 1.349)	(Ref.)	(0.515, 1.652)	(0.169, 1.608)
Physical activity						
Physically active						
All	1.000	0.708***	0.555***	1.000	0.801***	0.612***
	(Ref.)	(0.656, 0.764)	(0.477, 0.646)	(Ref.)	(0.729, 0.879)	(0.510, 0.734)
Urban	1.000	0.700***	0.544***	1.000	0.778***	0.599***
	(Ref.)	(0.645, 0.758)	(0.463, 0.640)	(Ref.)	(0.705, 0.859)	(0.493, 0.727)
Rural	1.000	0.838	0.716	1.000	1.082	0.747
	(Ref.)	(0.681, 1.031)	(0.470, 1.091)	(Ref.)	(0.835, 1.401)	(0.448, 1.245)

*Note*: Estimates were obtained using complex survey weights. 95% confidence intervals are in parenthesis. Regressions were separately estimated for each preventive behavior and behavioral risk factor for the full sample (i.e., All) and urban and rural sub‐samples. Each row presents results of a separate regression (unadjusted and adjusted). The adjusted odds were obtained by controlling for age, race and ethnicity, educational attainment, household income, marital status, employment status, and fixed effects.

*** *p* < 0.001

** *p* < 0.01

* *p* < 0.05.

Results for the different types of gynecologic cancer are presented in Table 4. Compared to respondents with no history of cancer, survivors of endometrial cancer had higher adjusted odds of getting a flu shot (AOR = 2.0, 95% CI: 1.5–2.7). Except for the ovarian cancer, the likelihood of having pneumonia vaccination was higher for survivors the other types of gynecologic cancer. While the adjusted odds of smoking were significantly higher (AOR = 4.0, 95% CI: 3.2–5.0) among survivors of cervical cancer, survivors of endometrial cancer had a significantly lower likelihood of heavy drinking (AOR = 0.4, 95% CI: 0.2–0.7). Lastly, the lower odds of physical activity among gynecologic cancer survivors were evident across all three types of gynecologic cancer.

**TABLE 4:** Unadjusted and adjusted odds ratios in favor of preventive measures and behavioral risk factors for gynecologic cancer types.

	No cancer	Unadjusted odds ratio			Adjusted odds ratio		
		Cervical	Endometrial	Ovarian	Cervical	Endometrial	Ovarian
Cancer screening							
Colorectal cancer							
All	1.000	1.081	2.102***	1.652*	1.249	1.533	1.370
	(Ref.)	(0.818, 1.430)	(1.476, 2.994)	(1.040, 2.623)	(0.905, 1.723)	(0.976, 2.406)	(0.784, 2.392)
Mammogram							
All	1.000	0.776	1.207	1.245	0.792	1.150	1.314
	(Ref.)	(0.572, 1.051)	(0.810, 1.797)	(0.741, 2.090)	(0.566, 1.107)	(0.738, 1.795)	(0.762, 2.266)
Vaccination							
Influenza							
All	1.000	0.928	1.994***	1.045	0.972	1.407*	0.854
	(Ref.)	(0.757, 1.138)	(1.501, 2.650)	(0.751, 1.455)	(0.760, 1.243)	(1.013, 1.954)	(0.591, 1.233)
Pneumonia							
All	1.000	1.477***	3.778***	1.551**	1.407**	1.979***	0.931
	(Ref.)	(1.208, 1.805)	(2.872, 4.970)	(1.118, 2.153)	(1.088, 1.820)	(1.343, 2.916)	(0.628, 1.381)
Tobacco/alcohol consumption							
Current smoking							
All	1.000	4.020***	0.807	1.349	3.115***	0.924	1.058
	(Ref.)	(3.244, 4.981)	(0.514, 1.266)	(0.867, 2.098)	(2.412, 4.024)	(0.548, 1.559)	(0.667, 1.676)
Heavy drinking							
All	1.000	0.986	0.363**	0.937	1.149	0.412*	1.080
	(Ref.)	(0.662, 1.468)	(0.186, 0.709)	(0.412, 2.133)	(0.757, 1.742)	(0.202, 0.840)	(0.467, 2.498)
Physical activity							
Physically active							
All	1.000	0.574***	0.552***	0.516***	0.710**	0.514***	0.524**
	(Ref.)	(0.467, 0.707)	(0.419, 0.725)	(0.363, 0.733)	(0.559, 0.902)	(0.363, 0.728)	(0.343, 0.801)

*Note*: Estimates were obtained using complex survey weights. 95% confidence intervals are in parenthesis. Regressions were separately estimated for each preventive behavior and behavioral risk factor for the full sample (i.e., All). Each row presents results of a separate regression (unadjusted and adjusted). Results for the “other cancer” group were not reported as they were identical to those reported in Table 3. The adjusted odds were obtained by controlling for age, race and ethnicity, educational attainment, household income, marital status, employment status, and fixed effects.

*** *p* < 0.001

** *p* < 0.01

* *p* < 0.05.

## 4 DISCUSSION

The results of this population‐based study indicate that adherence with colorectal cancer screening and pneumonia vaccination recommendations among gynecologic cancer survivors compares favorably with that of persons with no history of cancer. Adherence to breast cancer screening and influenza vaccination, on the other hand, were not different across the two groups. In addition, adherence with colorectal cancer screening test and influenza immunization recommendations among gynecologic cancer survivors is generally lower than that of survivors of other types of cancer.

In this study, 25.97% of gynecologic cancer survivors are current cigarette smokers and 5.08% are heavy alcohol drinkers, thus at higher risk of cancer recurrence, secondary malignancies, and other chronic diseases. Of note, though alcohol consumption does not directly impact the risk of ovarian and endometrial cancer,^22^ heavy alcohol use may increase the risk of cervical cancer recurrence.^23^ Gynecologic cancers include both smoking‐related cancer (cervical cancer) and non‐smoking‐related cancers (ovarian cancer, uterine cancer).^24^ Oncologists and primary care providers have important roles in encouraging cancer survivors to reduce their risk of serious illness or death through healthy lifestyle changes. The prevalence of cigarette smoking was especially high (35.49%) among gynecologic cancer survivors who live in rural areas.

Gynecologic cancer survivors in the current study were less likely to be physically active than persons without a history of cancer and having other types of cancer. Of note, as per data availability, the measure of physical activity analyzed in this paper, included leisure time physical activities only and did not take into account of any physical activities in the workplace. Further, the BRFSS data did not provide information on whether the recommended guidelines for weekly physical exercise were met. As such, this measure may not reflect the true rate of recommended physical exercise. However, given the data constraint it can be considered as a close proxy to the actual rate as used in other studies.^25^ Our results are consistent with those reported by Hawkins et al.^26^ in the Prostate, Lung, Colorectal, and Ovarian Cancer Screening trial who found that cancer survivors were less likely to engage in physical activity than those who were cancer free. As such, it is important to make women with gynecologic malignancies aware about the importance of physical activity.^4^

Adopting and maintaining healthy behaviors, however, is subject to some structural barriers at individual, social, and environmental levels.^27^ For example, lack of equipment or facility as well as time constraints are some barriers to physical exercise for cancer survivors.^28^ Additionally, perceived structural barriers among healthcare professionals impedes promotion of physical activity in cancer patients.^29^, ^30^ Psychosocial factors, such as lack of knowledge and advice, external motivation, and monitoring are other barriers of healthy eating and physical exercise among cancer patients.^31^ Addressing these structural barriers can significantly improve healthy behaviors among survivors of gynecologic cancers.

With respect to limitations, the results may not be generalizable to all gynecologic cancer survivors in the United States because of the low response rate in the BRFSS. In addition, misclassification bias may have occurred due to the use of self‐reported information. Misclassification refers to the classification of an individual, a value or an attribute into a category other than that to which it should be assigned. We did not perform single or multiple imputation to deal with missing data in the analysis. However, because the amount of missing data in BRFSS is small, this is unlikely to have resulted in any important bias.

We were limited by lack of data on temporality, including pre‐diagnosis behaviors. Behaviors such as smoking, alcohol consumption, and physical activity may change following a cancer diagnosis. Due to the cross‐sectional nature of the data, we were unable to assess any behavioral changes during pre‐ and post‐diagnosis phases. Furthermore, we did not have information on cancer characteristics (e.g., stage at diagnosis), phase of care, or treatment patterns, which could influence the preventive and risky health behaviors. Future research using appropriate longitudinal data may address these issues.

In conclusion, the results of this study have substantial implication for healthcare providers, members of the healthcare team, and researchers. A recent study reported a higher risk of cardiovascular diseases and obesity among gynecologic cancer survivors.^32^ As such, promotion of preventive health behaviors can play a critical role in improving health and wellbeing of women with gynecologic malignancies. Though adherence to certain cancer screening (i.e., colorectal cancer) and immunization (i.e., pneumonia vaccination) recommendations among gynecologic cancer survivors compared favorably with that of those with no history of cancer, the prevalence of smoking is markedly higher among gynecologic cancer survivors. Intervention studies are needed to identify effective ways to assist gynecologic cancer survivors to quit smoking and refrain from alcohol consumption. In addition, increased efforts should be made to make women with gynecologic malignancies aware of the health risk behaviors, emphasizing the adverse effects that behaviors like smoking could have on cancer treatments. In addition, the importance of physical activity, and the impact of preventive practices should be stressed to these patients by all members of the healthcare team.

## AUTHOR CONTRIBUTIONS

**Steven S. Coughlin:** Conceptualization (equal); writing – original draft (equal); writing – review and editing (equal). **Biplab Datta:** Formal analysis (equal); writing – review and editing (equal). **Justin X. Moore:** Writing – review and editing (equal). **Marlo Vernon:** Writing – review and editing (equal). **Martha Tingen:** Writing – review and editing (equal).

## Data Availability

This study was based upon anonymous, public use data provided by the Centers for Disease Control and Prevention.
